# A cooperative scheme for late leaf spot estimation in peanut using UAV multispectral images

**DOI:** 10.1371/journal.pone.0282486

**Published:** 2023-03-27

**Authors:** Tej Bahadur Shahi, Cheng-Yuan Xu, Arjun Neupane, Dayle Fresser, Dan O’Connor, Graeme Wright, William Guo

**Affiliations:** 1 School of Engineering and Technology, CQUniversity, Rockhampton, QLD, Australia; 2 Institute for Future Farming Systems, School of Health, Medical and Applied Sciences, CQUniversity, Bundaberg, QLD, Australia; 3 Department of Agriculture and Fisheries, Bundaberg, QLD, Australia; 4 Peanut Company of Australia, Kingaroy, QLD, Australia; Universidade Federal de Uberlandia, BRAZIL

## Abstract

In Australia, peanuts are mainly grown in Queensland with tropical and subtropical climates. The most common foliar disease that poses a severe threat to quality peanut production is late leaf spot (LLS). Unmanned aerial vehicles (UAVs) have been widely investigated for various plant trait estimations. The existing works on UAV-based remote sensing have achieved promising results for crop disease estimation using a mean or a threshold value to represent the plot-level image data, but these methods might be insufficient to capture the distribution of pixels within a plot. This study proposes two new methods, namely measurement index (MI) and coefficient of variation (CV), for LLS disease estimation on peanuts. We first investigated the relationship between the UAV-based multispectral vegetation indices (VIs) and the LLS disease scores at the late growth stages of peanuts. We then compared the performances of the proposed MI and CV-based methods with the threshold and mean-based methods for LLS disease estimation. The results showed that the MI-based method achieved the highest coefficient of determination and the lowest error for five of the six chosen VIs whereas the CV-based method performed the best for simple ratio (SR) index among the four methods. By considering the strengths and weaknesses of each method, we finally proposed a cooperative scheme based on the MI, the CV and the mean-based methods for automatic disease estimation, demonstrated by applying this scheme to the LLS estimation in peanuts.

## Introduction

Crop health is impacted by various abiotic and biotic stresses. Environmental factors such as drought, floods, and extreme temperatures can cause abiotic stress whereas the biotic stress can be caused by pathogens such as fungi, bacteria and nematodes [1]. Moreover, crop disease caused by biotic stress such as fungi and bacteria has an adverse influence on crop quality and productivity.

Early detection and identification of such diseases are beneficial for farmers and breeders as they can implement appropriate management strategies to minimise the impact of these diseases and prevent or mitigate possible yield losses. Since manual disease scouting is time-consuming and can introduce human bias [2], developing fast disease assessment methods with high detection accuracy would be much beneficial to farmers and farm management. Hence, researchers have been looking for fast plant phenotyping approaches such as image analysis [3] and remote sensing [4], which can be used for rapid disease monitoring and other plant traits such as crop yield [5], canopy coverage [6], leaf wilting [7] and plant density [8] assessment with high speed and accuracy [3].

Remote sensing can be an alternative approach for fast and unbiased disease scouting and measurement [9]. Here, the common information carrier is the electromagnetic (EM) light spectrum. The light spectrum is measured with various wavelengths such as visible, multispectral, and hyperspectral sensors [10, 11]. There is a trade-off between the accuracy of information capture and the cost of these sensors [12]. Recently, unmanned aerial vehicles (UAVs) have gained increasing attention from agricultural researchers to be used in related applications such as disease detection [13], plant health monitoring [14], and precise pesticide application [15]. The UAV has been a more common choice in precision agriculture because a) it has the flexibility of revisiting the field at flexible time intervals, and b) it can capture high-resolution imagery much closer to the plant in comparison to satellite imagery [16]. With such high-resolution images, automatic disease detection for various crops such as wheat yellow rust detection [17], peanut leaf wilt estimation [18], and tomato spot wilt disease estimation [19] has been reported in the literature.

There have been many studies on plant phenotyping using the Red-green-blue (RGB) [18], multispectral [20], and hyperspectral [17] sensors in UAVs. The RGB and multispectral sensors are widely used in precision agriculture. With such images, the vegetation indices (VIs) are derived to measure the plant traits such as disease score and yield estimation. A few studies have reported the successful application of disease estimation using UAV-based remote sensing [17–19]. In these studies, for plot-level data extraction, either a mean value of the vegetation index or the number of pixels below a certain threshold in a given plot was used to estimate the disease score. For instance, Patrick et al. [19] investigated the multispectral image-derived vegetation indices such as Normalized Difference Red Edge (NDRE) and Normalized Difference Vegetation Index (NDVI) for tomato spot wilt disease estimation in peanuts. They first extracted the vegetation index from five-band multispectral images captured with a Micasense Red-edge camera. Then, they determined a threshold to distinguish the healthy and disease pixel value in these vegetation indices (VIs) images. Finally, the number of pixels below or above the given threshold was taken as a predictor and disease percentage as a target variable for linear regression analysis. They achieved an *R*^2^ of 0.82 with NDRE during the late season of peanut growth. They selected the optimal threshold for each vegetation index manually which makes this approach less ideal for automation. In addition, some vegetation indices might not have a clear threshold for healthy and disease plot segmentation, which further impedes the use of this approach for such vegetation indices. Furthermore, the spectral band selection and vegetation index construction are also influential factors that determine what information is more important for crop health and disease estimation.

Given the limitation of these existing methodologies, we propose a cooperative scheme that combines our newly proposed methods with the existing mean-based method together for disease estimation. These methods share the potential advantage in implementation for automatic processing and decision-making. To demonstrate and validate the usefulness and effectiveness of this cooperative scheme, we chose peanuts as a study crop for our experiment. Peanut is an annual crop in Australia, mainly grown in Queensland. It takes around four to five months from planting to maturity for early-season maturing peanut varieties. It is important to monitor the various biotic and abiotic stresses on peanuts during the growing season to assure that the good quality and quantity of peanuts are optimised [18]. The two most common foliar diseases in peanuts grown in Queensland are late leaf spot (caused by *Phaeoisariopsis personata*) and leaf rust (caused by *Puccinia arachidis*) [21]. These are the most destructive diseases of peanuts in Australia [22]. A high pod loss due to late leaf spot (LLS) and leaf rust (LR) of up to 61-85% has been reported [23–25] under greenhouse conditions. Thus, a rapid, objective estimation of the LLS disease in peanuts could help farmers to manage the disease epidemic and increase the pod yield. Therefore, this paper aims to investigate the applicability of UAV multispectral images to estimate the LLS disease severity in peanuts using our proposed cooperative scheme.

The main **contributions** of our work are as follows.

(i) We investigated the relationship between multispectral imagery-based vegetation indices and LLS disease score at the late growth stages of peanuts.
(ii) We proposed two new measurement index (MI) and coefficient of variation (CV) derived from a UAV multispectral image to estimate the LLS disease score.
(iii) We compared the performance of the proposed MI-based and CV-based methods with the threshold and mean-based methods for LLS disease estimation. The results show that the MI-based method outperforms the other three methods in most of the chosen indices, except the simple ratio (SR) index where the CV-based method performed similar to or better than the MI-based method.
(iv) By considering the strengths and weaknesses of each method, we proposed a cooperative scheme for the selection of appropriate methods for disease detection and forecasting in different situations.

Our paper is organized as follows. Section “Background information and image processing” introduces the crop study area, manual data collection, UAV image collection, and correlation analysis. The two popular existing methods and the two new methods proposed in this study are presented in the section “Methods for disease detection and estimation”, using the imagery featured selected from the previous section. The accuracy assessment matrices are discussed in the section “Evaluation matrices”. The experimental results are further discussed in the section “Results and discussion”, along with the cooperative scheme. Section “Conclusion and future works” concludes our work and presents further recommendations.

## Background information and image processing

**Fig 1:**
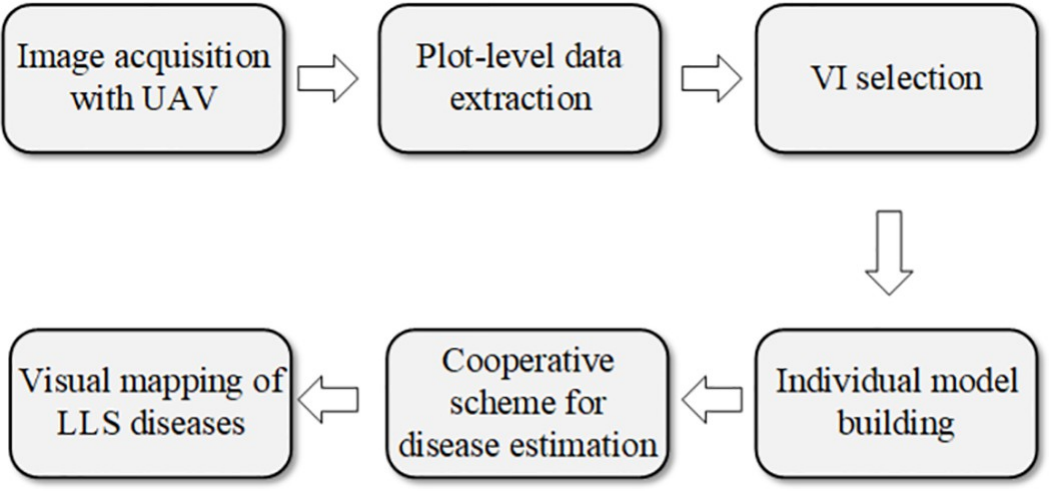
A block diagram showing the high-level workflow used in this study.

The overall block diagram of the proposed framework for late leaf spot (LLS) disease estimation on peanuts is shown in Fig 1. This framework consists of six steps: UAV image acquisition, plot-level data extraction, VI selection, individual model building, applying cooperative scheme for disease estimation and visual mapping of LLS disease. The discussion of each activity on the proposed pipeline is provided in the following sections.

### Crop study area

**Fig 2:**
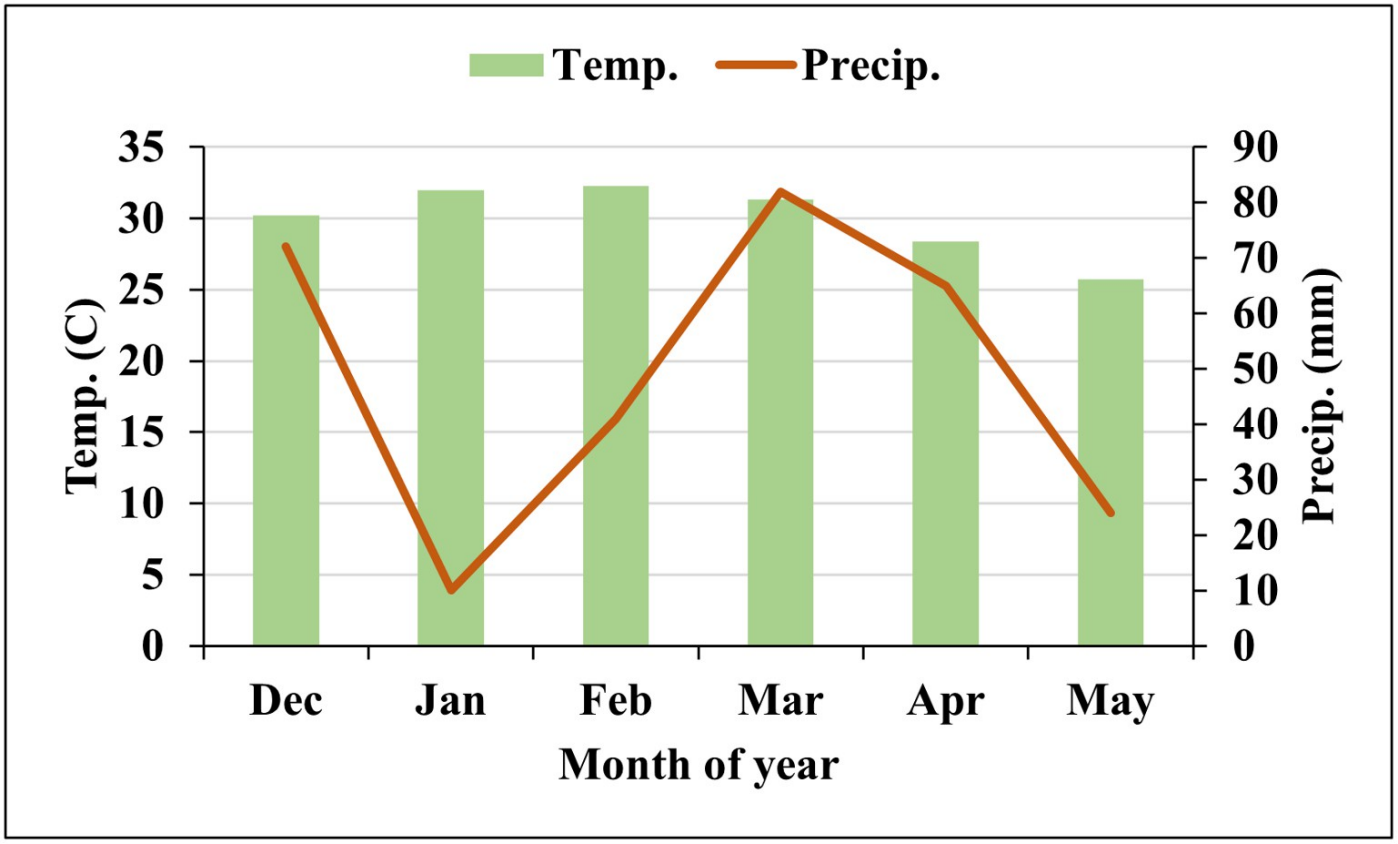
The temperature and precipitation of the study area during the experiment period (15/12/2018- 31/05/2019). Note that Temp. and Precip. denote the monthly average maximum temperature and total precipitation respectively.

The field data were collected from the Queensland Department of Agriculture and Fisheries, a research facility in Bundaberg, Queensland, Australia. The regional climate was categorized as temperate with an annual average maximum temperature of 28.5^*c*^ and annual precipitation of 320.0 mm for 2019 [26]. During the experimental period (Dec 2018 to May 2019), the rainfall in December was high (70.00mm) and it again reached 80mm in March and slowly decreased after. However, the temperature was mostly around 32.5^*C*^ (Feb, 2019) to 25.5^*c*^ (May, 2019) throughout the period (Fig 2). A peanut field consisting of 72 peanut breeding plots with twenty-four peanut genotypes and three replications of each, was planted on 19^*th*^ December 2018.

### Manual disease data collection

The manual assessment of disease rating was performed using a late leaf spot severity scale of 1-9 where 1 represents no disease (0% defoliation) and 9 represents 100% defoliation as described by Singh and Oswalt [27]. The manual disease assessment was performed on the 10^*th*^ of April 2019, which is 112 days after plantation (DAP). One experienced scouter of peanut diseases did all scoring to keep consistency of the rating. The manual LLS score dataset of 72 plots (where each plot has two peanut lines) had a mean LLS score of 4.77 and a standard deviation of 1.16.

### Image acquisition with UAV

**Fig 3:**
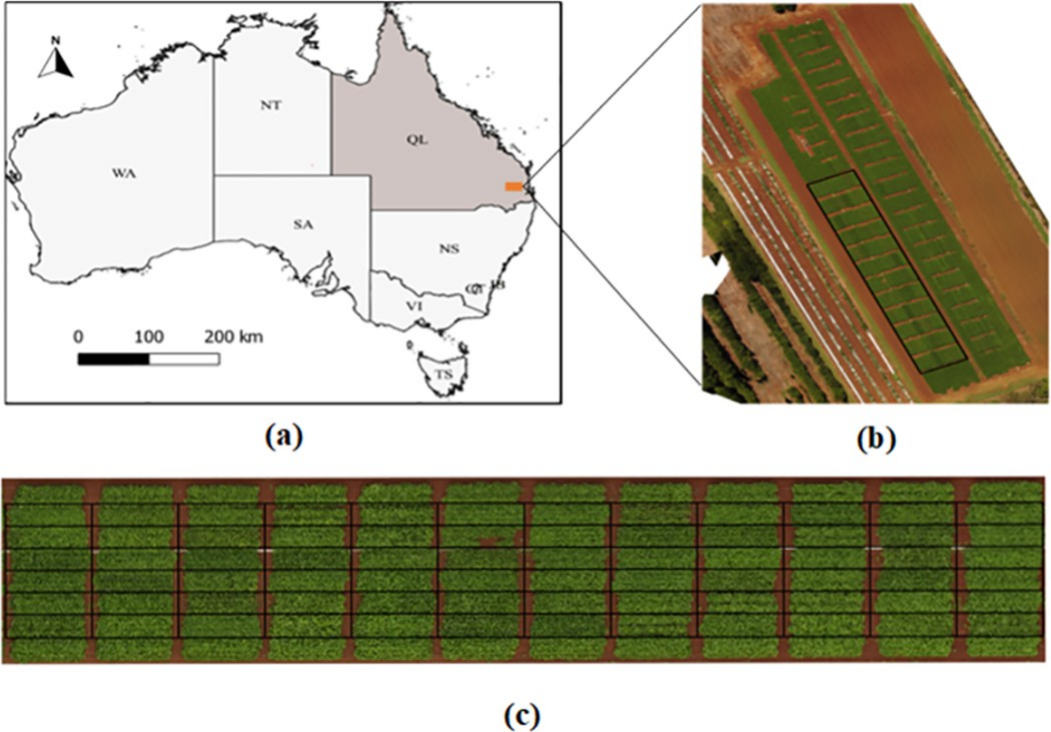
Study area map a) Field location in Australian Map. Note that the shapefile for this map is obtained from (https://www.diva-gis.org/gdata), b) RGB Orthomosaic of whole UAV trial c) Peanut field with 72 peanut breeding plots used in this study. Note that the black boxes layered over the images represent the Region of interest (ROI).

**Fig 4:**
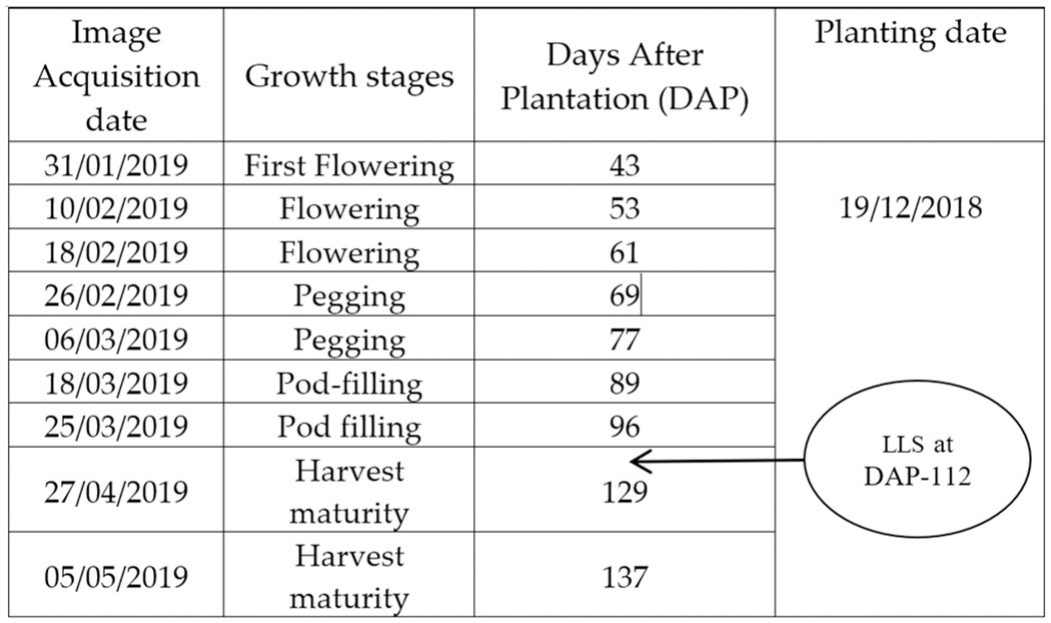
UAV images acquisition date of peanut trial.

We utilized a Phantom 3 Advanced multi-rotor drone (DJI, Shenzhen, China) with an integrated MicaSense RedEdge sensor (Micasense, Seattle, WA, USA) to acquire peanut field images at various growth stages. The images were acquired at a height of 40 meters above the ground and with a parallel camera CCD angle to the ground. The side and forward overlap of 60% and 90% were maintained in all drone flights while capturing aerial images, which generated a satisfactory performance of image stitching. The reflectance measurement with the multispectral camera included five spectral bands: Red (630-690nm), Blue (460-510nm), Green (545-575nm), Near-Infrared (820-860nm), and Red-edge (712-722nm). Each sample data was geo-referenced in the World Geodetic System (WGS) 1984 datum, Universal Transverse Mercator (UTM) Zone 55 projection. Six Ground Checkpoints (GCP) were surveyed and marked with a Real-time Kinematic (RTK), Global Positioning System (GPS) (Leica Geosystems CS20 controller plus GS14 antenna, Hexagon, Madison, AL, USA) for registering ground data with multispectral images, which provides a spatial error of less than 2cm across the field of the study area (Fig 3). Fig 4 reports the dates of multispectral image acquisition and the manual disease data measurements.

### Plot-level data extraction and vegetation indices

The overall plot-level data extraction process included the following steps. Initially, the acquired images in each UAV flight were transferred to a computing platform running the most popular software package, Pix4Dmapper (Pix4D S. A. Prilly, Switzerland), which is based on computer vision and photogrammetry methods. The automated processing template ‘Ag Multispectral’ included in the software package was used to orthorectify and mosaic the collected images. Second, once the orthomosaic was generated, it was uploaded to the quantum geographic information system (QGIS) software [28] to extract the area of interest by specifying the co-ordinate of a boundary point of the field. Third, the extracted peanut field was cropped and rotated to align peanut breeding plots perpendicular to the plane. Finally, the individual plot shape-file using an open-source R package- FIELDimageR [29] was created to divide a whole field into individual plots, consisting of coordinates points of individual plots. A sample plots division is shown in Fig 3, where rectangle boxes are overlayed on the RGB orthomosaic image.

The further pre-processing of five band multispectral images was carried out to remove the soil pixels and the edge effect on the orthomosaic. The soil pixels were filtered out using a filter mask defined in Eq 1 with a Green Red Vegetation Index (GRVI), which classifies the pixel into soil vs plant using a threshold value [29].

$$\begin{array}{c} GRVI=\frac{\left(G-R\right)}{\left(G+R\right)} \end{array}$$ (1)

where if *GRVI* ≤ 0.2 a pixel was masked out as a soil pixel; otherwise the pixel was considered as a plant pixel.

For edge effect reduction, we used buffering inside the polygon while extracting plot-level data. We considered the masked buffer of −0.1*m* in each plot polygon while extracting the plot pixel values.

We produced 12 vegetation index (VI) images using the five-band multispectral GeoTIFF images of the peanut field. The VIs selected were widely used in literature for plant growth monitoring [36], disease detection [18, 19] and yield estimation [33, 37]. We utilized the reflectance values from five spectral bands: Red (630-690nm), Blue (460-510nm), Green (545-575), Near-infrared (820-860nm), and Red-edge (712-722nm) to derive the vegetation indices [38, 39]. The formulas to calculate each vegetation index derived from these spectral bands are listed in Table 1.

**Table 1:** The list of vegetation indices used for LLS monitoring in this study. Note that *R*, *G*, *B*, *NIR* and *RE* represent the spectral bands: Red (630-690nm), Blue (460-510nm), Green (545-575), Near-Infrared (820-860nm), and Red-edge (712-722nm) respectively.

Vegetation index	Formula	Application	Crop	Reference
NDVI	$\frac{\left(NIR-R\right)}{\left(NIR+R\right)}$	Leaf wilting monitoring	Peanut	[30]
DVI	*NIR* − *R*	Disease detection	Wheat	[31]
SR	$\frac{NIR}{RE}$	Disease detection	Wheat	[19]
GNDVI	$\frac{\left(NIR-G\right)}{\left(NIR+G\right)}$	Brown spot and blast disease detection	Rice	[32]
NORM2	$\frac{NIR}{NIR+R+G}$	Yield estimation	Maize	[33]
NORM3	$\frac{R}{NIR+R+G}$	Yield estimation	Maize	[33]
NDRE	$\frac{\left(NIR-RE\right)}{\left(NIR+RE\right)}$	Disease detection	Peanut	[19]
SAVI	$\frac{\left(1.5*(NIR-R)\right)}{\left(NIR+R+0.5\right)}$	Brown and blast detection	Rice	[32]
PSRI	$\frac{\left(R-G\right)}{\left(RE\right)}$	Bacterial leaf blight monitoring	Rice	[34]
REVI	$\frac{RE}{\left(NIR+RE+G\right)}$	Spot wilt detection	Peanut	[19]
GVIRE	$\frac{\left(G-RE\right)}{\left(G+RE\right)}$	Spot wilt detection	peanut	[19]
LAI	$\frac{3.618*2.5*(NIR-R)}{\left(NIR+6*R-7.5*B+1\right)}-0.118$	Yield estimation	Rice	[35]

### Correlation between VI and LLS rating

**Fig 5:**
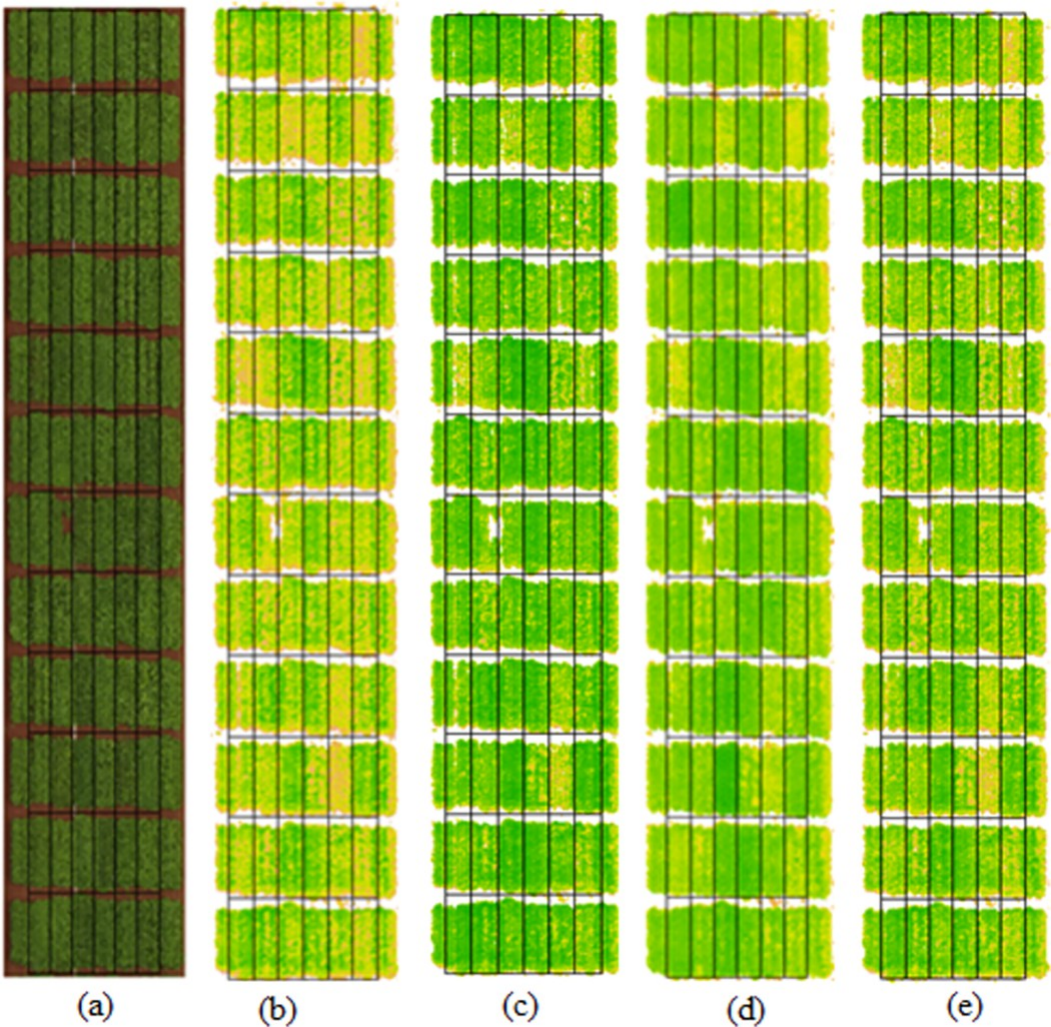
The RGB orthomosaic of the peanut field acquired at DAP-96 used in this study a), and its corresponding shape-file overlayed on the VI images b) DVI, c) SAVI, d) NDRE e) LAI.

Each vegetation index (VI) was further analysed to find its relationship with the disease score. We calculated the Pearson correlation to measure the strength of the linear relationship between each VI and LLS score. For this, we first extracted pixel values for each spectral band from a five-band UAV orthomosaic. Second, we used the formula listed in Table 1 to derive a vegetation index image. Finally, the vegetation index image was used to extract the mean VI value of each peanut plot by using the polygons as shown in Fig 5.

While observing the Pearson’s correlation (Table 2), we noticed that six vegetation indices, namely NDRE, DVI, SAVI, SR, LAI, and REVI have a high correlation (|*R*| > 0.68) at the pod-filling growth stage (DAP-96), while other vegetation indices have a lower correlation. Hence, we selected these six vegetation indices for further consideration. It is worth noting that the REVI index has a positive correlation with the LLS score whereas the other five VIs (NDVI, DVI, SAVI, SR, and NDRE) have a negative correlation. It might be due to the spectral ratio of a red-edge with NIR and Green bands being more discriminative for the LLS score than the other combination of RGB and near-infrared bands.

**Table 2:** The correlation coefficient between the LLS at DAP-112 and VI images taken in various peanut growth stages. Note that ^a,b^ represents the correlation at 0.01 and 0.05 level of significance respectively.

Vegetation index	DAP-43	DAP-53	DAP-61	DAP-69	DAP-77	DAP-89	DAP-96	DAP-129	DAP-137
NDVI	-0.15	-0.17	-0.25	-0.17	-0.21	-0.29	−0.65^*b*^	−0.57^*a*^	0.06
DVI	-0.05	-0.35	-0.33	-0.35	-0.32	−0.42^*a*^	−0.80^*b*^	−0.57^*b*^	-0.12
SR	-0.15	-0.21	-0.29	-0.23	-0.27	−0.30^*a*^	−0.68^*b*^	−0.58^*b*^	0.04
GNDVI	-0.01	-0.03	-0.23	-0.16	-0.21	-0.11	−0.61^*b*^	−0.50^*b*^	0.11
NORM2	-0.00	0.22	-0.01	-0.00	-0.00	-0.15	-0.11	-0.09	-0.28
NORM3	0.12	-0.12	0.09	-0.08	0.05	0.03	0.38	0.51^*a*^	-0.25
NDRE	-0.05	-0.09	-0.19	-0.21	-0.27	-0.22	−0.68^*b*^	−0.52^*a*^	0.12
SAVI	-0.07	-0.35	-0.33	-0.32	-0.31	−0.41^*a*^	−0.79^*b*^	−0.58^*b*^	-0.10
PSRI	0.21	0.28	0.04	0.12	0.14	0.28	0.34	0.57^*a*^	0.08
REVI	0.06	0.10	0.18	0.22	0.28	0.24	0.69^*b*^	0.52^*a*^	-0.12
GVIRE	-0.12	-0.02	0.27	0.04	-0.00	-0.14	0.20	0.42	-0.06
LAI	-0.10	-0.35	-0.32	-0.34	-0.32	−0.43^*a*^	−0.80^*b*^	−0.58^*b*^	-0.09

## Methods for disease detection and estimation

### Threshold-based method

**Fig 6:**
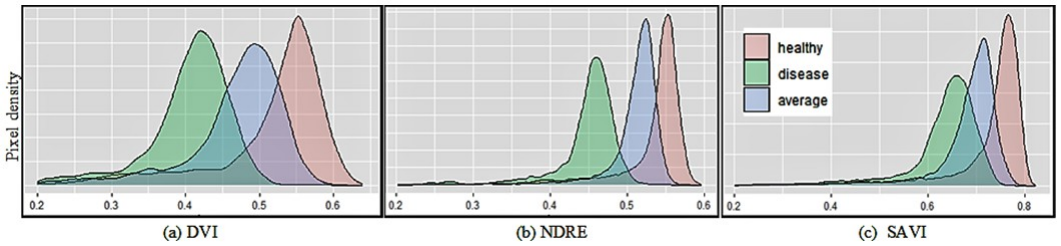
The sample plot distribution of three kinds of plots: Healthy, average, and highly diseased plots. Note that the density plots are drawn by taking an average of five randomly chosen plots for each category of plots from the image taken at DAP-96. Note that the plots with disease scores (1-2), (3-5) and (6-9) are considered healthy, moderately diseased and severely diseased plots respectively for this illustration.

**Fig 7:**
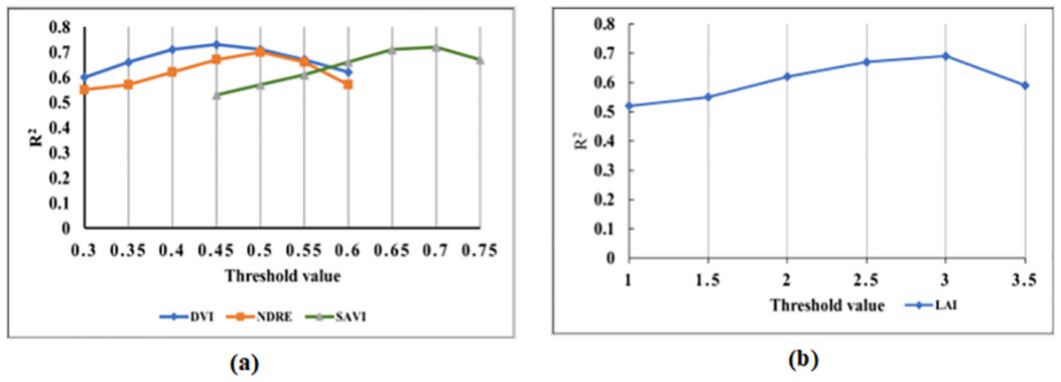
The change in a coefficient of determination (*R*^2^) with the selected threshold value for three vegetation indices DVI, NDRE, and SAVI a) and LAI b) with UAV image taken at DAP-96.

To establish a threshold for diseased and healthy plots segmentation, we followed a similar procedure explained by Patrick et al. [19]. We plotted the pixel density distributions for both the diseased and healthy plots by taking an average of five healthy (LLS score between 1-2), moderately diseased (LLS score between 3-5) and severely diseased plots (LLS score between 6-9) selected randomly. The average distribution plots are shown in Fig 6. While observing such plots, we noticed that the pixel distributions in each healthy, moderately diseased, and severely diseased plots are visually separable for all indices to some extent. Therefore, the threshold for each vegetation index that separates these plots optimally needs to be found empirically. To determine the optimal threshold, we used a sensitivity analysis which was based on choosing an optimal threshold value from a range of possible values by comparing the evaluation metrics such as coefficient of determination (*R*^2^). Here, we used the proportion of pixels above the threshold as an independent variable (x) and the LLS score as a dependent variable (y) for correlation analysis. We choose the proportion of pixels above the threshold instead of a raw count of pixels above the threshold in a single peanut plot because we filtered out the soil pixels from each plot which resulted in a different number of total plant pixels in each plot. Also, we didn’t consider pixel proportion below the threshold as suggested in [19]. For instance, optimal threshold selection for vegetation index DVI, NDRE, LAI and SAVI using sensitivity analysis is shown in Fig 7. Since the threshold value is different for different vegetation indices and must be identified empirically for each index, applying this method for disease score estimation is more tedious and challenging.

Once the threshold is selected, the linear regression model for disease score estimation was built with pixel proportion above the threshold as an independent variable, genotype as a control variable and disease score as a dependent variable.

### Mean-based method

This is the widely used method for plot-level data extraction in the UAV image processing pipeline [17, 18]. Here, the average pixel value for each plot was extracted from each vegetation index (VI) image (Note that the soil pixels and edge effects were removed before the vegetation index images generation). Then, we applied linear regression with the average VI value as an independent variable, genotype as a control variable and manual LLS score as a dependent variable. This method is straightforward as compared to the threshold-based method as it does not require any threshold value.

### Coefficient of variation or CV-based method

Referring to Fig 6, the spatial distribution of pixels in healthy and diseased peanut plots is significantly different where the healthy plots have a high and narrower pick in comparison to diseased plots. Since the coefficient of variation (CV) as defined in Eq 2 measures the relative dispersion of pixels around the mean, we used CV as an independent variable to estimate LLS using linear regression. Unlike the mean-based method that considers only one single feature (the mean value *μ* or a single point value in a wide range) from the simple statistics, the CV method takes the two key statistic features, the mean (*μ*) and the standard deviation (*σ*) from the simple statistics, into account, which still does not require determining any tunable parameters such as threshold value.

$$\begin{array}{c} CV=\frac{\sigma }{\mu } \end{array}$$ (2)

CV has the ability to relatively enlarge the statistical difference between the two means for the diseased and healthy plots. For example, assuming *μ* = 0.3 and *σ* = 0.2 for the diseased plot and *μ* = 0.6 and *σ* = 0.1 for the healthy plot, the ratio between the mean for the diseased plot and the mean for the healthy plot is 0.3/0.6 = 0.5. Meanwhile, the CV for the diseased and healthy plots should be 2/3 and 1/6 respectively. The ratio between the CV for the diseased plot and the CV for the healthy plot is 4. This indicates that by using CV the relative difference between the diseased and healthy plots is 8 times higher than that using the means. However, the quantitative influence of this relative ratio needs to be further studied in the future.

### Measurement index (MI)-based method

Our proposed measurement index (MI) is also based on the two key statistical features (mean and standard deviation) from the simple statistics and defined in Eq 3, to gauge the pixel distribution variation among the healthy and diseased peanut plots.

$$\begin{array}{c} MI=(\mu -\alpha \sigma ) \end{array}$$ (3)

Where *μ*, and *σ*, represent the mean and standard deviation of the pixel distribution in a peanut plot and *α* is a constant which represents the effect of standard deviation on MI.

**Fig 8:**
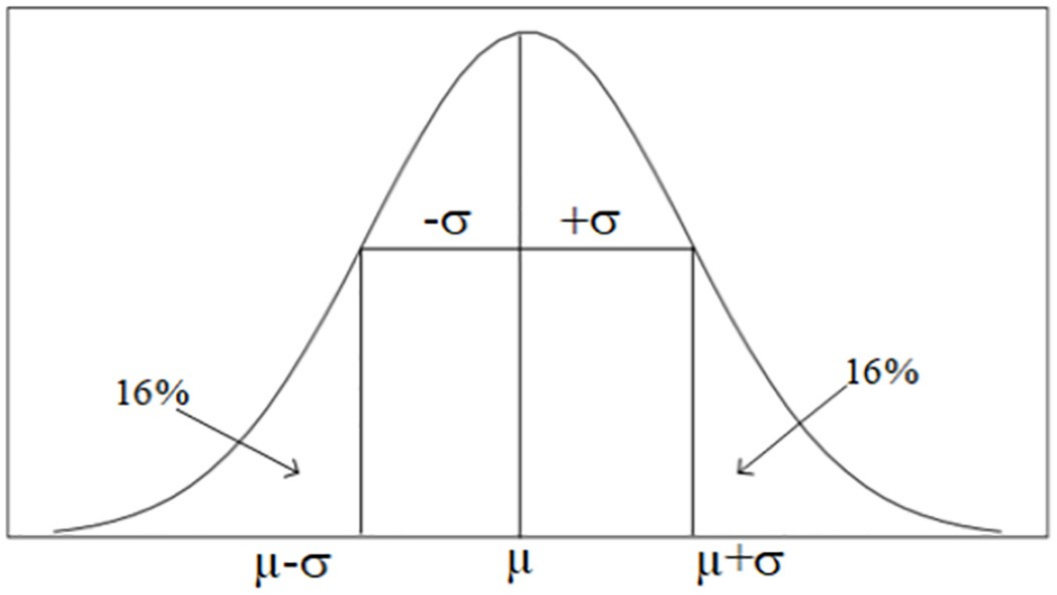
The normal distribution plot with a mean (*μ*) and standard deviation (*σ*).

In statistics, if a density plot is normally or near normally distributed, MI points to the left- wing of the curve where its accumulated density to the mean is about 16% when *α* = 1 (Fig 8). Under the assumption of *α* = 1, MI can also further enlarge the statistical difference between the two means for the diseased and healthy plots through the ratio between the healthy and diseased plots, the opposite way to the CV-based method through the ratio between the diseased and healthy plots. As a qualitative example, assuming *μ* = 0.3 and *σ* = 0.2 for the diseased plot and *μ* = 0.6 and *σ* = 0.1 for the healthy plot, the ratio between the mean for the healthy plot and the mean for the diseased plot is 0.6/0.3 = 2. Meanwhile, the MI for the healthy and diseased plots should be 0.5 and 0.1 respectively. The ratio between the MI for the healthy plot and the diseased plot is 0.5/0.1 = 5. This indicates that by using MI the relative difference between the healthy and diseased plots is 2.5 times higher than that using the means. This also indicates that the CV and MI methods are not overlapping with each other; rather, they are complementary to each other from opposite mechanisms. If one misses the target of detection, the other may be able to pick it up. Similar to the CV-based method, the quantitative influence of this relative ratio needs to be further studied in the future.

**Fig 9:**
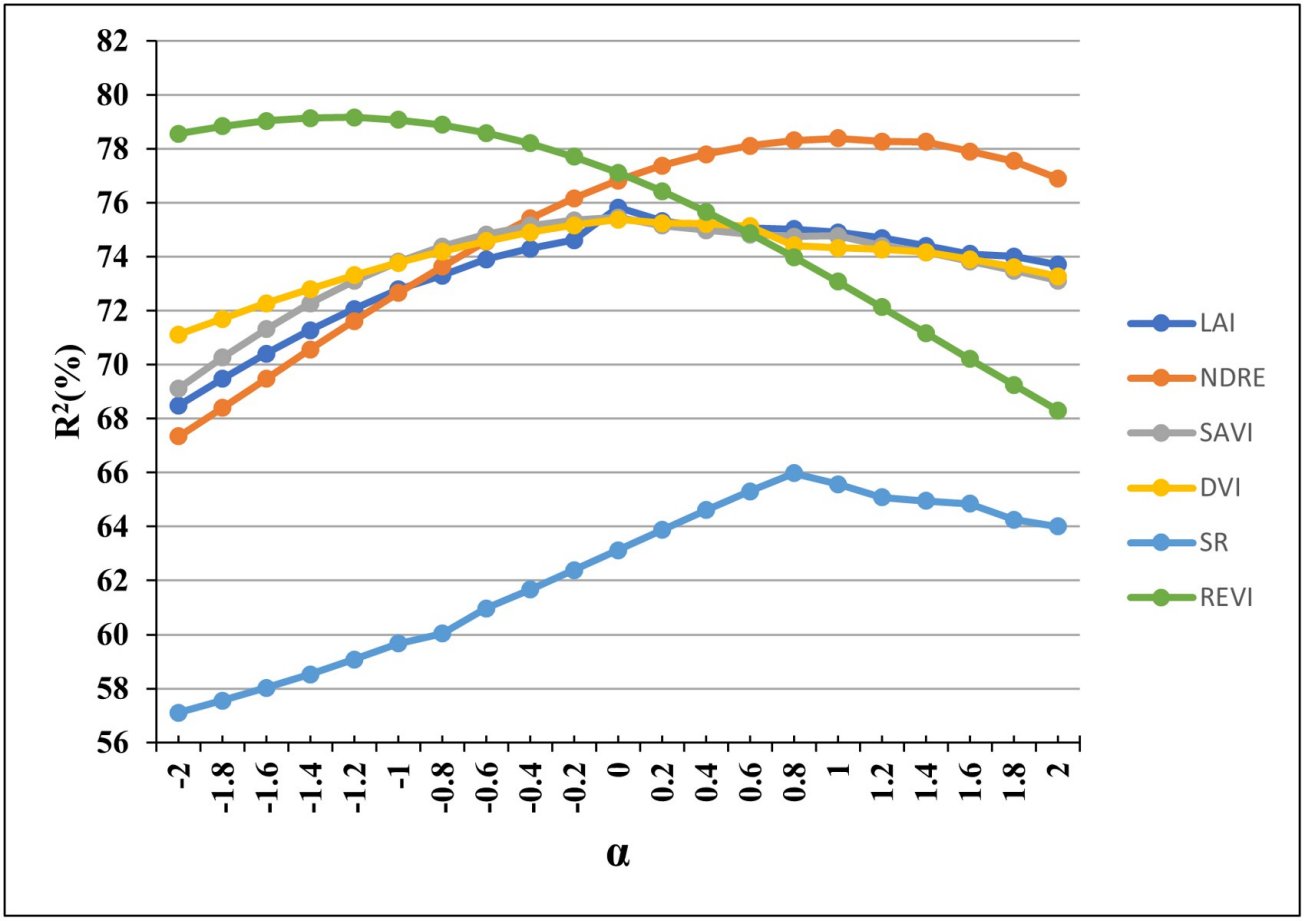
The convergence curve for the optimal value of the parameter (*α*) in MI-formula for six selected Vegetation Indices (VI).

The constant factor (*α*) may be variable to different indices. We explore whether an optimum value for the constant factor exists for each of the six VIs selected in this study. Hence, a sensitivity analysis for *α* from -2 to 2 (since the ±3*σ* represents only about 0.13% density on normal distribution, its effect is negligible on MI) was conducted and the coefficient of determination was measured using regression analysis between the MI values and the manual disease scores. We utilized the drone image taken at DAP-96 because the vegetation indices were highly correlated with the image at this growth stage. The experimental results are plotted in Fig 9.

From Fig 9, we see that the optimal (*α*) value for LAI, SAVI, and DVI, is about zero, which indicates that the MI-based method for LAI, SAVI and DVI would be almost the same to the mean-based method. The optimal (*α*) value for NDRE and SR is around 0.8 to 1.2, which indicates that the MI-based method would be better in distinguishing the disease status by NDRE and SR with a value smaller than the mean value by about one standard deviation. The optimal (*α*) value for REVI is negative around –1.5, which indicates that the MI-based method would be better in distinguishing the disease status by REVI with a value greater than the mean value by about 1.5*σ*. Note that REVI is positively correlated with the manual LLS score (Table 2), in contrast to the other five selected VIs.

## Evaluation metrics

The widely used parameters, the coefficient of determination (*R*^2^), Mean Absolute Error (MAE), and Relative Root Mean Square Error (RRMSE) are employed to measure the performance of LLS estimation methods. The MAE (Eq 4) and RRMSE (Eq 5) measure the absolute error and the error percentage respectively. The coefficient of determination measures the explanation of variation by the regression analysis model [40].

$$\begin{array}{c} MAE=\frac{\sum_{i=1}^{n}\left|y_{i}-z_{i}\right|}{n} \end{array}$$ (4)

$$\begin{array}{c} RRMSE=\frac{100}{\bar{z}}\sqrt{\frac{1}{n}\sum_{i=1}^{n}{\left(y_{i}-z_{i}\right)}^{2}} \end{array}$$ (5)

Where *y*_*i*_ and *z*_*i*_ are the estimated and measured values respectively. The $\bar{z}$ represents the mean of the measured values. We used LOOC (Leave one out cross-validation) strategy to validate the regression models [41]. In this strategy, one sample is kept for verification and k-1 samples are used for model training. The average accuracy of all k-runs was taken as the final model accuracy. This process was performed in the R-Caret package [42].

## Result and discussion

The assessment metrics of the experimental results from applying the threshold, mean, CV and MI-based regression models to the two multispectral images DAP-96 and DAP-129 are summarized in Tables 3 and 4. These two multispectral images were chosen because the manual LLS disease score was taken on DAP-112 which is closer to these two stages (refer to Fig 4).

**Table 3:** The performance comparison of four methods (threshold-based, mean-based, CV-based and MI-based) on six selected vegetation indices for UAV-derived images taken before (DAP-96) manual LLS rating (y). Note that the best thresholds chosen for NDRE (0.50), SAVI (0.7) and DVI (0.45), LAI (3.0), SR (25.0), REVI (0.22) for the threshold-based method (Ref. to Fig 7).

VIs	Threshold-based method			Mean-based method			MI-based method			CV-based method		
	*R* ^2^	MAE	RRMSE	*R* ^2^	MAE	RRMSE	*R* ^2^	MAE	RRMSE	*R* ^2^	MAE	RRMSE
NDRE	0.70	0.41	10.68	0.76	0.36	9.48	0.78	0.35	9.16	0.60	0.49	12.42
SAVI	0.72	0.38	10.27	0.75	0.37	9.76	0.75	0.37	9.88	0.64	0.45	11.79
DVI	**0.73**	0.37	9.85	0.75	0.37	9.77	0.75	0.36	9.75	0.65	0.45	11.62
LAI	0.69	0.41	10.92	0.74	0.37	9.87	0.74	0.37	9.80	0.65	0.45	11.62
SR	0.58	0.48	12.69	0.63	0.45	11.96	0.65	0.45	11.45	**0.66**	0.44	11.44
REVI	0.70	0.40	10.71	**0.77**	0.36	9.43	**0.79**	0.33	8.32	0.52	0.53	13.62

**Table 4:** The performance comparison of four methods (Threshold-based, Mean-based, MI-based and CV-based) on selected six vegetation indices for UAV-derived image taken after (DAP-129) manual LLS rating. Note that the best thresholds chosen for NDRE (0.45), SAVI (0.67) and DVI (0.45), LAI (2.6), SR (27.0), RDVI (0.55), REVI (0.24) for threshold-based method.

VIs	Threshold-based method			Mean-based method			MI-based method			CV-based method		
	*R* ^2^	MAE	RRMSE	*R* ^2^	MAE	RRMSE	*R* ^2^	MAE	RRMSE	*R* ^2^	MAE	RRMSE
NDRE	0.54	0.49	13.33	0.59	0.48	12.58	0.60	0.48	12.41	0.56	0.51	12.98
SAVI	0.52	0.52	13.63	0.61	0.48	12.21	0.63	0.47	11.97	0.57	0.49	12.78
DVI	0.52	0.52	13.53	0.61	0.47	12.20	0.63	0.46	11.86	0.56	0.50	13.07
LAI	0.51	0.52	13.65	0.61	0.48	12.21	0.62	0.47	11.99	0.58	0.49	12.74
SR	0.53	0.50	13.45	0.56	0.50	12.93	0.60	0.47	12.33	0.63	0.45	11.90
REVI	0.53	0.53	13.32	0.59	0.48	12.49	0.59	0.48	12.55	0.52	0.53	13.57

### Observations with the threshold-based method

The regression results with threshold-based method showed that the correlation between pixel proportion derived from each vegetation index image and LLS rating was high at DAP-96 while it weakened at the late harvest maturity stage or DAP-129 (Tables 3 and 4) on two flight dates before (March 25^*th*^) and after (April 27^*th*^) manual scoring (April 10^*th*^). A similar pattern was reported by Patrick et al. [19] for spot wilt disease estimation using the threshold-based method. The disease condition appeared to exacerbate soon after early April.

While comparing the strength of the vegetation index to estimate the LLS disease score using the threshold-based method (Table 3), the DVI had the highest *R*^2^ (0.73) and lowest error (9.85% RRMSE). The second-best performing VI was SAVI with *R*^2^ of 0.72. The vegetation indices such as SR and LAI have the least *R*^2^ and higher errors (MAE and RRMSE).

### Observations with the mean-based method

Since the threshold-based method required identifying the best threshold for each vegetation index to separate the healthy and disease pixels, it was difficult to find the best threshold for some vegetation indices as there was no clear separation in pixel value that segments the image into healthy and disease classes. However, the mean-based method was simple to implement and the results showed that it is more efficient than the threshold-based method as seen in Tables 3 and 4.

From Table 3, we observed that the majority of the vegetation indices have a coefficient of determination (*R*^2^) above 0.74 while only the vegetation index (SR) has a minimum *R*^2^ (0.63) in comparison to the threshold-based method. Similarly, it produced comparable results with the MI-based method while being inferior in a few vegetation indices such as NDRE and SR. However, it can be seen that the mean-based method outperformed the threshold-based method in all vegetation indices but it is still inferior to the MI-based method in all vegetation indices (Tables 3 and 4).

### Observations with the CV-based method

The CV-based method is also straightforward to implement as it does not include any extra parameters which need to be found empirically. While looking at Tables 3 and 4, we notice that the CV-based method achieved the highest *R*^2^ and lowest error for SR among the four methods, followed by the MI-best method. However, its performance on other VIs were the worst among the four methods.

### Observations with MI-based method

The MI-based method showed more promising results in comparison to the other three methods in estimating the LLS disease score using the six vegetation indices on DAP-96 (refer to Table 3). As expected, the MI-based method returned the best performance for NDRE and REVI, and slightly below the CV-based method for SR, and the same performance as the mean-based method for SAVI, DVI and LAI, on which the MI-based method takes zero value for the constant factor *α*. Hence, the MI-based method could incorporate the mean-based method in these circumstances, making it the standout leader ahead of the mean-based method and CV-based method except for SR.

It seemed the accuracy of all methods when applied to the later image at DAP-129 was reduced slightly as shown in Table 4. There might be two possible reasons for this. First, the vegetation indices calculated at a later stage carry less spectral information as the crops have reached their maturity stage. Second, the disease might have exacerbated to its tip and the crop canopy might have been damaged fatally, which affected the spectral information furthermore. However, it is essential to have a multiple manual disease score rating to confirm the effect of such parameters on the model but unfortunately, it was not available in our cases. Nevertheless, the MI-based method was the leader over the other three methods for all VIs except SR remained the same, so did the CV-based method for SR while applying it to image at DAP-129.

Furthermore, the comparative results revealed that the MI-based method is best for LLS estimation at DAP-96 while using vegetation indices NDRE,and REVI. It is interesting to note that these two VIs included both NIR and red-edge (RE) spectral bands which are the most important spectrum for monitoring peanut vegetation health. However, they demand the extra spectral ability of the sensor beside the low cost-RGB. Since, the vegetation indices SAVI, DVI and LAI converted the MI-based methods equivalent to mean-based method (where the optimal value of (*α*) is zero in the MI-formula), the single MI-based method can be utilized for such vegetation indices as well. Furthermore, the SAVI, DVI and LAI indices do not require a red-edge spectral band and depend only on RGB and NIR bands, so four bands of multispectral sensors are sufficient. The improvement in prediction accuracy with the MI-based method over the mean-based method also suggests that the disease score is not only affected by the mean VI value but also reflected by standard deviation. Hence, consideration of standard deviation in measurement index might be more effective in other disease estimation as well.

### Relationship between the MI-based and CV-based methods

**Fig 10:**
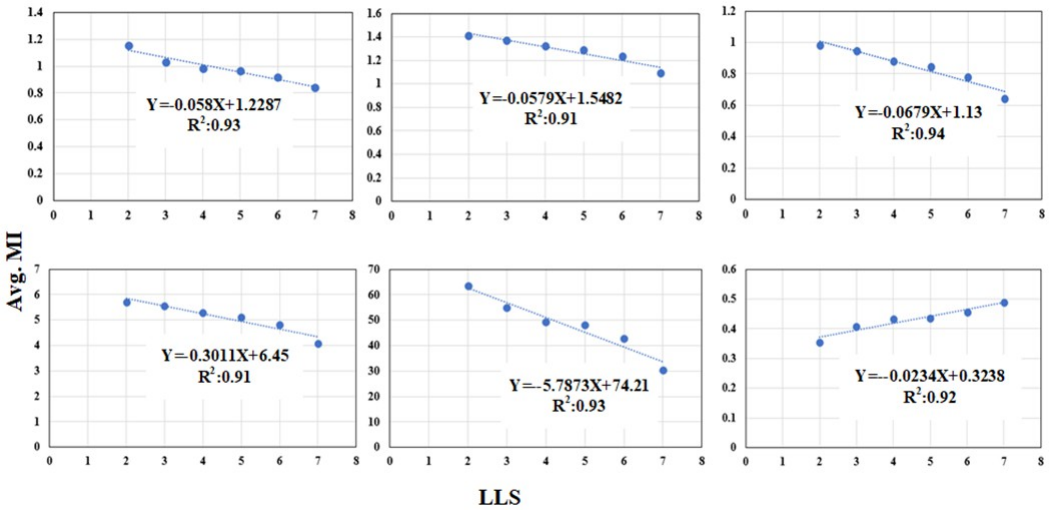
The linear association between LLS rating and Average MI derived for vegetation index a) NDRE, b) SAVI c) DVI d) LAI e) SR and f) REVI. Note the MI is averaged based on the LLS manual rating measured on DAP-112.

**Fig 11:**
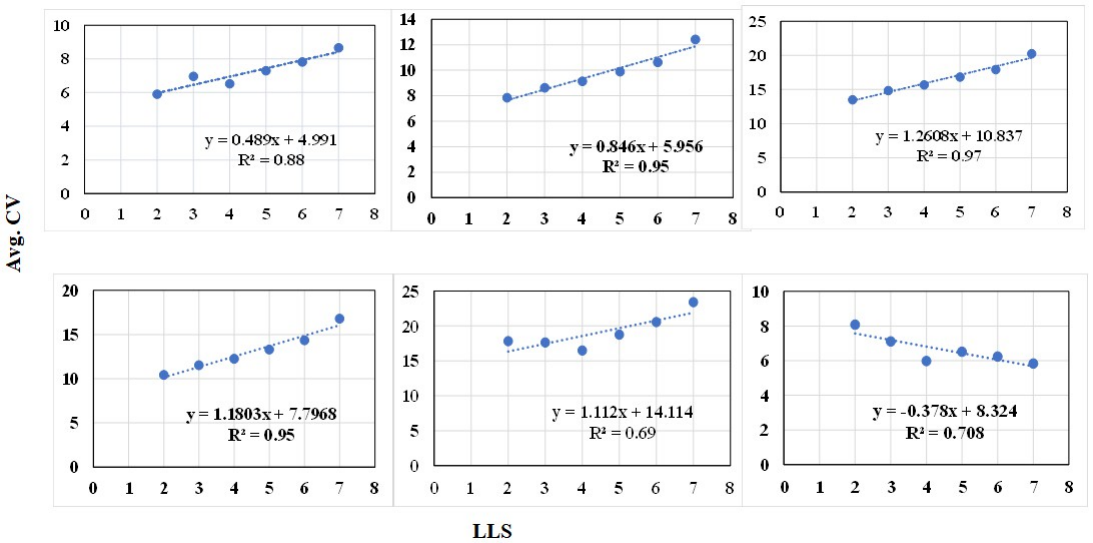
The linear association between LLS rating and Average CV derived for vegetation index a) NDRE, b) SAVI c) DVI d) LAI e) SR and f) REVI. Note the CV is averaged based on the LLS manual rating measured on DAP-112.

To evaluate the cooperative relationship between proposed MI-based and CV-based methods for LLS disease monitoring, we further evaluated them by taking the average measurement index (MI) and CV value based on the measured LLS rating score. The relationship plots are shown in Figs 10 and 11. While comparing these relationship plots, it is evident that the MI-based method and CV-based method have a complementary nature in terms of trending with LLS score. For instance, the relation between MI and LLS is negative for NDRE, SAVI, DVI, LAI and positive for SR whereas these trends are inversed between CV and LLS score.

### The regression models between MI and LLS

**Fig 12:**
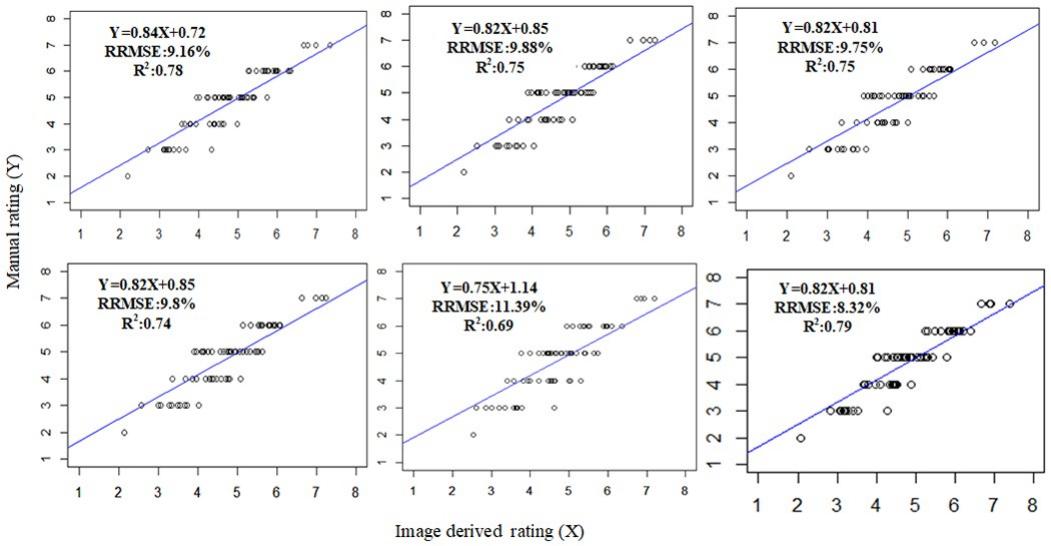
The correlation plots of the relationship between vegetation index a) NDRE, b) SAVI c) DVI d) LAI e) SR and f) REVI derived LLS rating based on MI at DAP-96 and Manual rating measured on DAP-112.

Since the MI-based method is the standout leader among the four methods for all six VIs (second best for SR), we can use the regression between the MI value at DAP-96 and the LLS scores on DAP-112 for early LLS estimation. The regression plots for the six VI are shown in Fig 12. Strong correlations exist between the MI and LLS scores. It would be the best to use the MI and LLS scores from the same day. But, the UAV images were not collected on DAP-112. For the purpose of early disease detection and estimation, these regression models might be more useful.

### The cooperative scheme for LLS disease estimation using UAV multispectral images

**Fig 13:**
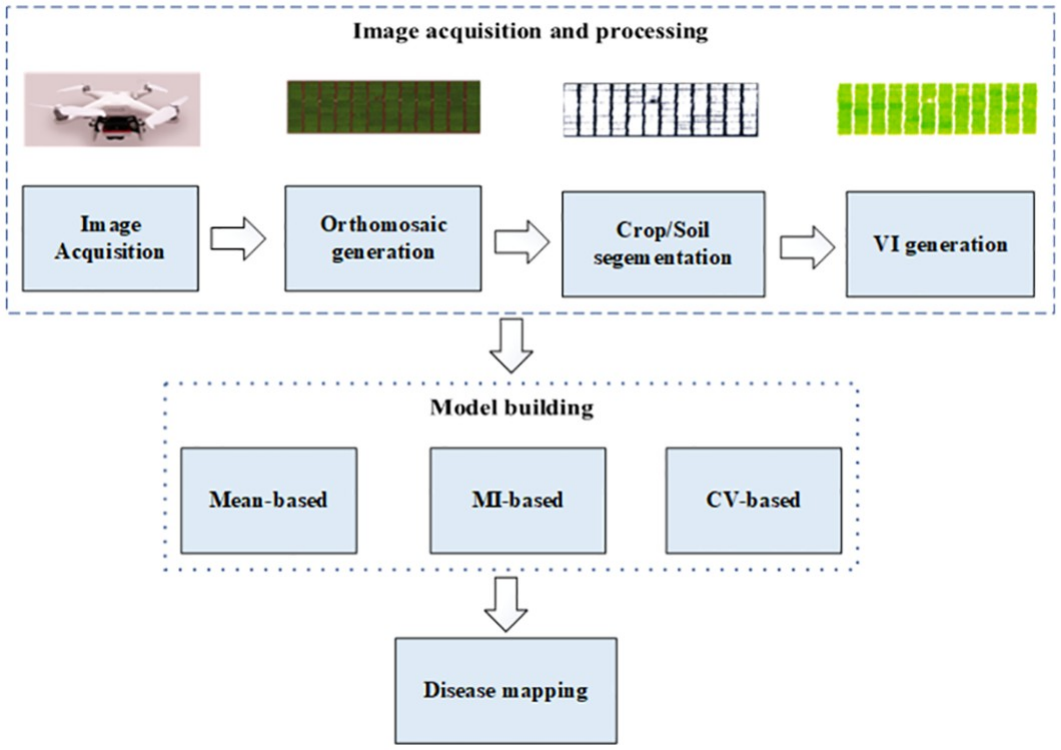
A high-level flowchart of proposed cooperative scheme for automatic disease estimation using UAV multispectral images.

Based on the observations and discussions on the performances of the four methods on disease detection and estimation above, considering the assessment metrics, simplicity of computing and automatic decision-making, and the complementary of among the methods, we propose a cooperative scheme for automatic disease estimation using UAV multispectral images illustrated by the overall flowchart shown in Fig 13. This scheme consists of three main components: UAV image acquisition and processing, model building and disease estimation, and disease mapping. This scheme incorporates the MI-based and CV-based methods, as a pair of complementary models built on the key statistical indicators of the mean and standard deviation, for automatic decision-making on disease estimation, with the mean-based method for its simplicity in computing and automatic decision-making as the second best performer just behind the MI-based method in most cases. Unfortunately, the threshold-based method is left out due to its manual nature of choosing a threshold for each of many chosen VIs and inferior performance compared to both the MI-based and Mean-based methods. The procedure of our cooperative scheme is listed in Procedure 1.

**Procedure 1** Cooperative scheme for LLS disease estimation

1: Acquire crop field image with UAV and multispectral camera.

2: Pre-process UAV multispectral images.

3: Separate crop from soil using GRVI index.

4: Generate any of 12 VI images and select a VI.

5: **if** *VI* ∈ [*SAVI*, *DVI*, *LAI*] **then**

6:  Choose mean-based methods for disease estimation.

7: **else if** *VI* ∈ [*NDRE*, *REVI*] **then**

8:  Choose MI-based methods for disease estimation.

9: **else if** *VI* ∈ [*SR*] **then**

10:  Choose CV-based methods for disease estimation.

11: **end if**

### An example of applying cooperative scheme

**Fig 14:**
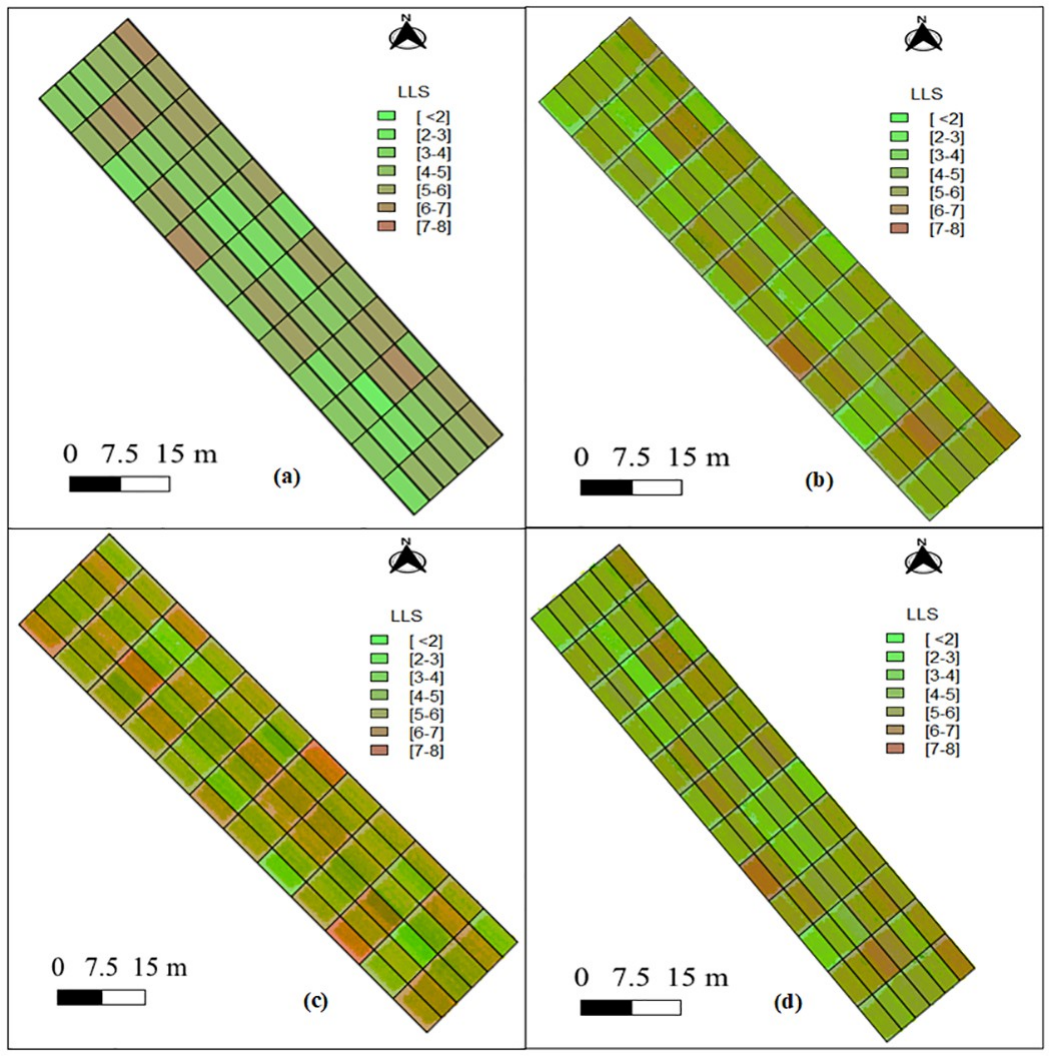
The visual mapping of late leaf spot (LLS) in a peanut field of 72 plots a) actual LLS score, and LLS score estimated with b) MI-based method (using REVI) c) CV-based method (using SR), e) mean-based method (using DVI).

Following the Procedure 1, we apply the proposed cooperative scheme to DAP-96 UAV images with the regression model from the three methods (Mean, CV and MI-based). The visual mapping of the LLS disease score over the 72 peanut plots is shown in Fig 14. The MI-based method has the more consistent prediction and mapping of LLS disease score. It is visually similar to the actual LLS score while comparing Fig 14(a) and 14(b). Among the other two methods, CV-based (refer to Fig 14(c)) method has mapped the majority of the peanut plots as highly diseased (dark red) while the actual LLS map (refer to Fig 14(a)) has very few highly disease plots (LLS score of 6-7 and 7-8). However, the visual mapping obtained with mean-based method (Fig 14(d)) is similar to the LLS mapping with MI-based method. This is expected as the quantitative performance of these two methods is very close to each other (79% of *R*^2^ for MI-based method and 75% of *R*^2^ for mean-based method).

## Conclusion and future works

This paper investigated the linear relationship between twelve widely used vegetation indices (VIs) with LLS disease ratings on peanuts. The correlation analysis of vegetation indices with LLS shows that the six vegetation indices namely, NDRE, SAVI, DVI, LAI, SR and REVI are highly correlated with LLS disease rating at the late growth stage of peanut (DAP-96) while the correlation is almost similar and lower for all vegetation indices at the earlier growth stage (i.e. DAP-77 or early stages).

The performance of the two alternative methods, namely, CV and MI-based methods proposed in this study were evaluated against that of the mean and threshold-based methods in estimating the LLS disease score using those six selected VIs for UAV images over different peanut plots. The results indicated that the MI-based method outperformed other three methods in all indices in most cases except the case where SR with the CV-based method achieved the best performance over other methods. For indices SAVI, DVI and LAI that depend on RGB and NIR bands without involving a red-edge spectral band, the MI-based method becomes the mean-based method. Hence, it seems that the MI-based method could represent the mean-based method for all selected indices both involving or not involving a red-edge spectral band. However, the MI-based method seemed variable with images/crops and indices in terms of choosing the optimal value for *α*. It seemed both NDRE and REVI involving a red-edge spectral band had a robust performance with |*α*| in a range of 0.8-1.4 although more experiments will be required on understanding the influence of *α* on the performance of the MI-based method in the future.

Overall, the threshold-based method seems redundant compared with other three methods in disease detection through analysing the UAV images. Considering the strengths and weakness of each of the MI-based, CV-based and mean-based methods, the following cooperative scheme seems an optimal solution for disease estimation through analysing the UAV images.

(a) For indices SAVI, DVI and LAI without involving a red-edge spectral band, either the mean-based method or the MI-based method can be selected;
(b) For indices NDRE and REVI involving a red-edge spectral band, the MI-based method should be selected;
(c) For SR involving a red-edge spectral band, the CV-based method should be the first choice, backed up by the MI-based method.

This study has two limitations. First, it only considered the vegetation index for regression analysis while the inclusion of other stress and multi-index decision-making could enhance the performance. Second, the manual LLS disease score was recorded only at the late growth stage which prevents us from investigating how early this method can be applied for disease estimation. As an extension of this work, advanced data-driven methods such as convolutional neural networks (CNN) can be investigated for building disease estimation models. Also, in choosing optimized parameters for prediction, simulated annealing [43] may be incorporated with CNN or other prediction methods. Furthermore, the disease progress can be monitored by recording the manual disease score at multiple times and conducting corresponding UAV flights to capture the canopy information. This will give an opportunity to make a time series analysis of disease progression and can be helpful to design an early disease detection model.
